# Brachiocephalic Vein Injury Leading to Massive Hemothorax Following the Insertion of a Tunneled Dialysis Catheter Requiring Surgical Intervention for Repair: Report of a Rare Case

**DOI:** 10.7759/cureus.32396

**Published:** 2022-12-11

**Authors:** Sultan Sarwar Parvez, Saikat DasGupta, Reazul Hoque, Mozibul Haque

**Affiliations:** 1 Cardiac Surgery, Square Hospitals Ltd., Dhaka, BGD; 2 Cardiothoracic Surgery, Square Hospitals Ltd., Dhaka, BGD; 3 Cardiac Anesthesiology, Square Hospitals Ltd., Dhaka, BGD

**Keywords:** median sternotomy for hemothorax, catheter induced hemothorax, tunneled dialysis catheter, right sided hemothorax, brachiocephalic vein injury

## Abstract

Dialysis catheters are commonly used tools for patients requiring hemodialysis. A dilator with a large caliber is used to insert such a catheter, which in turn can result in serious vascular injury leading to rare complications like hemothorax. Common treatment options for such vascular injuries comprise surgical repair by thoracotomy or video-assisted thoracoscopic surgery (VATS) and endovascular repair. We herein report a case of a brachiocephalic vein injury and massive right-sided hemothorax following the insertion of a tunneled dialysis catheter in the right internal jugular vein, treated successfully by our team at Square Hospitals Ltd.

## Introduction

The tunneled dialysis catheter (TDC) is a catheter used for hemodialysis, and it is tunneled under the skin [1]. Commonly available tunneled catheters are of two types: a) cuffed and b) non-cuffed. The non-cuffed catheters are usually used for emergency situations, for a shorter duration of time (up to one week). On the other hand, cuffed catheters are usually used for a duration longer than three weeks when an arterio-venous (AV) fistula has been created but is not yet matured for hemodialysis. Often, the preferred location for catheter access is the right internal jugular vein, for its straighter course into the superior vena cava and a reduced risk of malposition and thrombosis [2-4]. During the insertion of this catheter, a stiff dilator is used, which sometimes can lead to serious vascular injuries ultimately causing massive hemothorax, hypotension, cardiac arrest, or even death [5-7]. This usually happens in those patients who are on hemodialysis for a longer period of time and patients with central venous occlusive pathology (due to adhesion from repeated lines) [1,2,8]. The common treatment options are surgical repair by open thoracotomy, video-assisted thoracoscopic surgery (VATS) [5-7], median sternotomy, or endovascular repair [9,10]. In this case report, the right brachiocephalic vein injury led to massive hemothorax in the right pleural cavity, which was successfully managed through median sternotomy.

## Case presentation

A 53-year-old male patient with end-stage renal disease (ESRD), hypertension, and type 2 diabetes mellitus with ischemic dilated cardiomyopathy was admitted to our hospital. This time, he was admitted with complaints of respiratory distress, features of uremia, volume overload, with raised serum creatinine level. The patient’s previous medical records showed that he received two to three sessions of dialysis for acute kidney injury (AKI) two months prior to admission to our hospital, with non-compliance with treatment for the last couple of months. The attending nephrologist decided to restart hemodialysis with TDC (Quinton Permcath). Before starting the procedure, informed written consent was taken from the patient after proper counseling. All vital signs were monitored.

**Figure 1:**
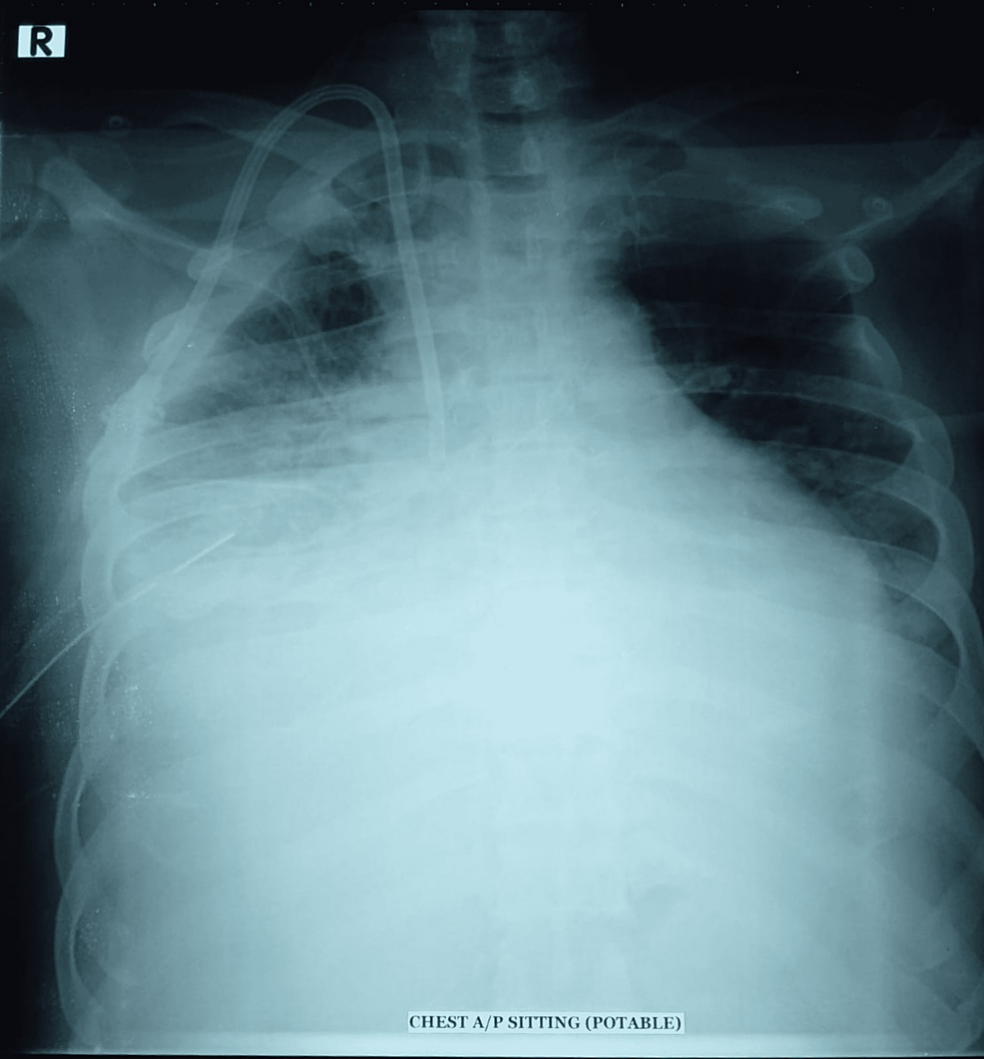
X-ray chest AP view showing right-sided pleural effusion with intercostal chest drain and dialysis catheter in-situ AP: anteroposterior

Under local anesthesia, the right internal jugular vein (RIJV) was punctured using ultrasound guidance. As the free-flowing venous blood was obtained through the puncture needle, a guidewire was introduced through the puncture needle. The overlying skin was then again infiltrated with lidocaine for subcutaneous tunneling. A 12 Fr dilator along with its sheath was then inserted with the help of the previously placed metallic guidewire. After the dilatation, the tunneled dialysis catheter was introduced followed by the removal of the sheath. The performing physician felt some unusual resistance during catheter placement. The patient complained of mild pain, along with shortness of breath and discomfort in the right side of the chest during this procedure. Within hours of the incident, the chest pain was gradually increasing and was later radiating toward the back. The patient ultimately developed tachycardia (heart rate 100-110 beats/minute) and became tachypnic. Immediate post-procedural chest X-ray (CXR) anteroposterior view showed whitening of the mid and lower lobes of the right lung field (Figure 1).

**Figure 2:**
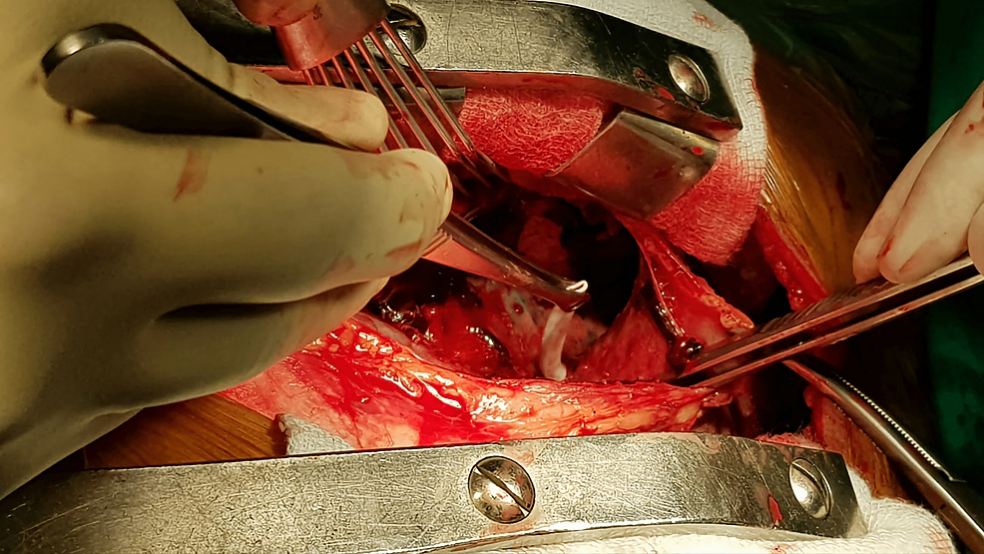
Peri-operative picture showing catheter in the right pleural cavity

**Figure 3:**
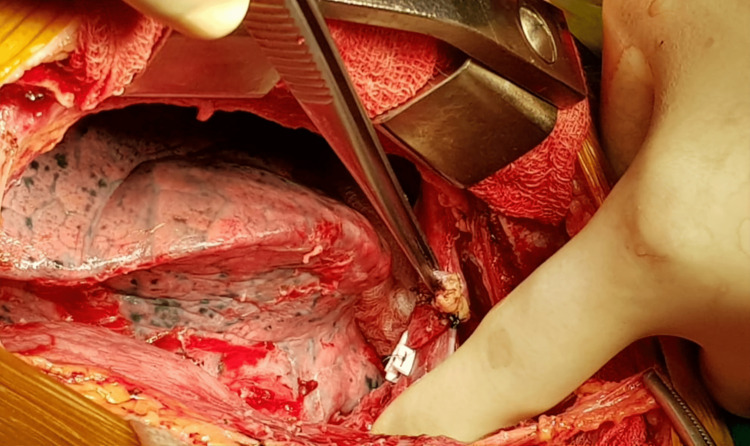
Peri-operative picture showing repaired catheter exit point

**Figure 4:**
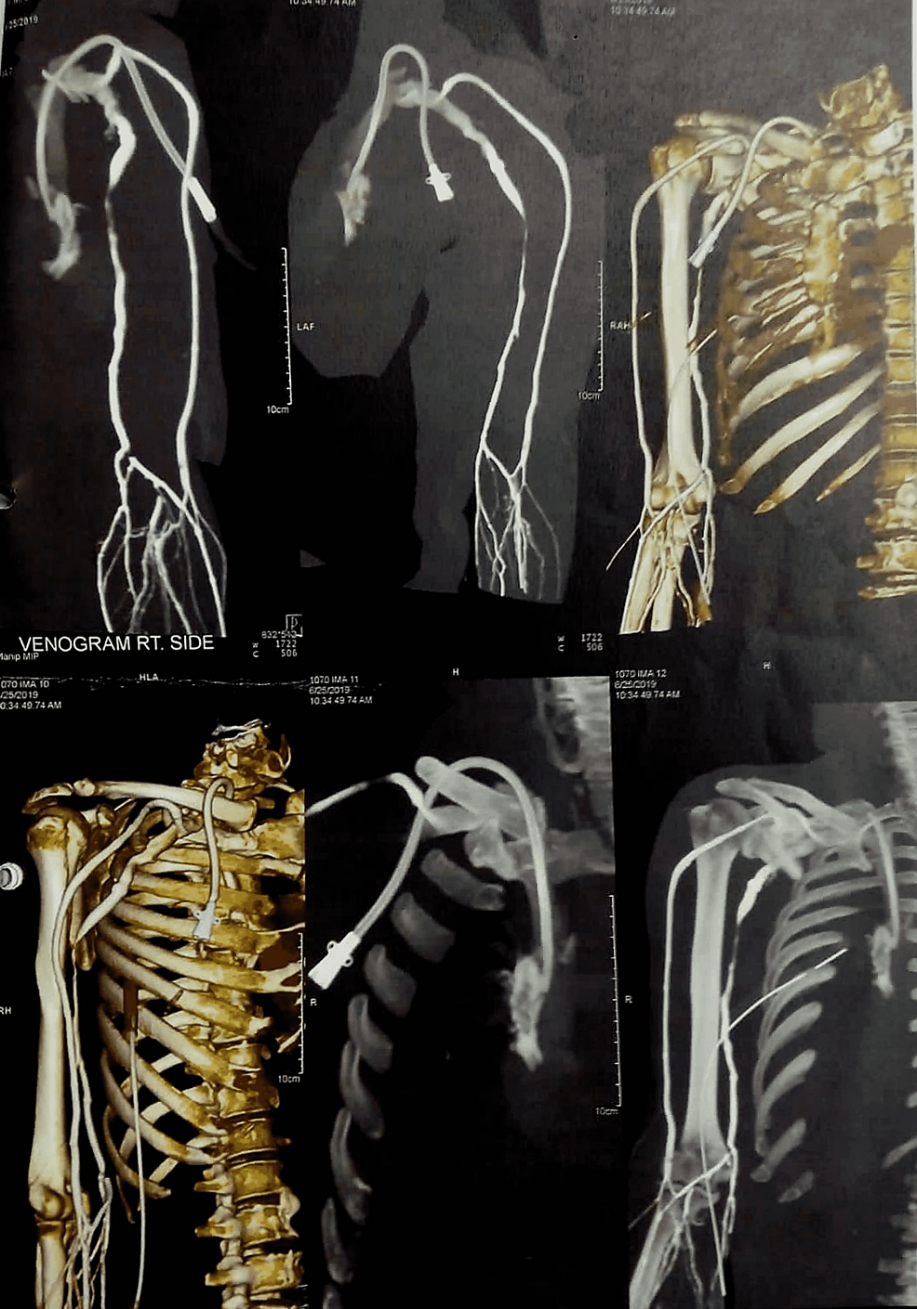
CT venogram showing catheter in situ

**Figure 5:**
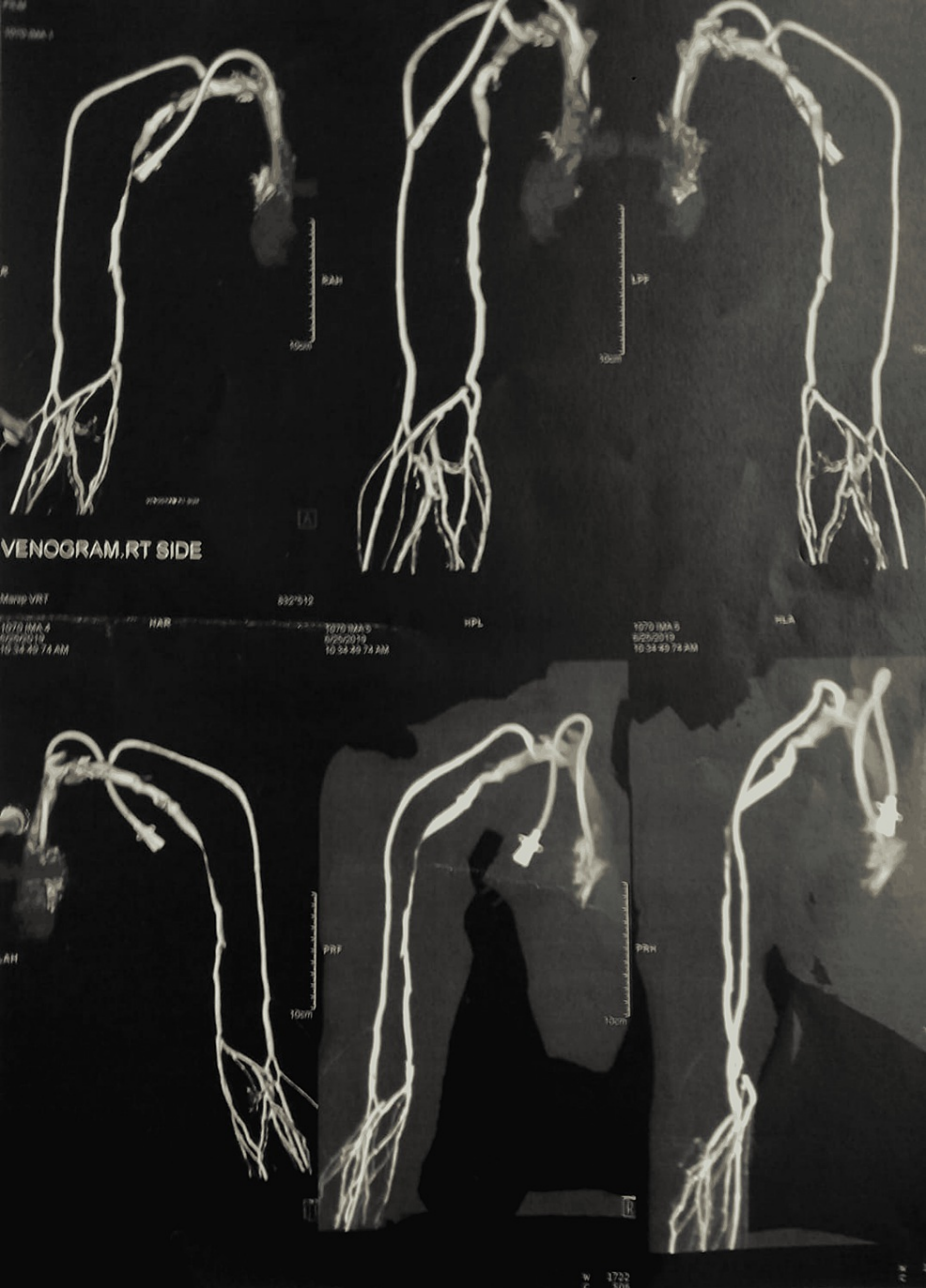
CT venogram showing catheter in situ

An urgent tube thoracostomy was done to relieve the symptoms. About 500 ml of blood was evacuated following tube thoracostomy. A femoral catheter (FC) was inserted electively, as he needed urgent dialysis. He was then shifted to the ICU for further monitoring but he became more unstable hemodynamically. Resuscitation started with a crystalloid solution, along with oxygen by face mask. The patient’s routine blood work along with blood grouping and cross-matching was done. Cardiovascular consultation was done and a follow-up CXR was done to evaluate right-sided hemothorax. CT venogram of the upper right limb including the right side of the chest revealed that the catheter entered the right brachiocephalic vein through its anterior wall behind the medial part of the right clavicle and then pierced the posterior wall about 1 cm below the entry point. The catheter was seen in the mediastinum posterior to SVC. No definite filling defect was seen in the axillary and subcutaneous veins. After the evaluation of the patient, an elective median sternotomy was done under general anesthesia in the supine position. The right-sided mediastinal pleural was opened to track the injury site in the right pleural cavity. A large hematoma along with clotted blood was found in the right pleural cavity, which was evacuated and the catheter tip was then found at the right pleural cavity piercing through the posteroinferior part of the right brachiocephalic vein (Figures 2-3). The brachiocephalic vein injury was repaired with 5-0 polypropylene and the dialysis catheter was removed externally. After proper hemostasis, the chest was closed in layers leaving a chest drain tube in situ (Figures 4-5). The patient was then extubated the next morning with stable hemodynamics. His postoperative hospital stays were uneventful. The chest tube was removed on the third postoperative day and the patient was transferred to the cabin. On the fifth postoperative day, the patient was discharged from the hospital without any complications.

## Discussion

The tunneled dialysis catheter is an established primary dialysis access for many patients requiring hemodialysis. In various places, particularly in less developed countries, many patients receive dialysis through this catheter throughout their arterio-venous fistula maturation period [11]. The catheter insertion and dilatation procedure may sometimes lead to grave complications like hematoma, pneumothorax, subcutaneous emphysema, thoracic duct injury, arterial puncture, hemothorax, pericardial tamponade, mediastinal hematoma, and catheter-induced infection [12,13]. Hemothorax, in most published reports, resulted from arterial injury and rarely from venous injury by needle or catheter [2,12,13]. It can very rarely occur from dilator injury [2]. The dilator, because of its stiff texture, can cause vascular injury more severe than a needle or catheter [2]. Sudden perforation of the brachiocephalic vein-SVC junction may initiate cardiac tamponade resulting in sudden hemodynamic decompensation [4,14].

Arik et al. [15], in one of their published reports, showed a case of mediastinal hematoma after placement of a left subclavian venous catheter for hemodialysis purposes in an end-stage renal failure patient [4]. The guidewire most probably caused bleeding that resulted in mediastinal hematoma by perforating the subclavian vein and ultimately led to the death of the patient. The vascular complication could possibly be explained by forced manipulation of the dilator or guidewire against tissue resistance [4]. Some factors that might increase the risk of central venous perforation by dialysis catheters are: a) left internal jugular vein (LIJV) catheterization, as the distance from the LIJV to the right atrium is more than the right and the catheter has to pass through the left brachiocephalic vein and the superior vena cava [5]; b), obesity [16], and c), larger diameter catheters if placed from the left side [17]. The route of dilator insertion is equally important, as the right internal jugular vein (RIJV) follows a medial course while draining to the superior vena cava and thus the dilator should be advanced along the course of the vein. Moreover, while advancing the dilator, undue force should be avoided.

The ultrasound-directed technique is advocated during the intervention, to avoid the above-mentioned complications. Ultrasound can delineate the vessel tree and vascular luminal patency, thus guiding the site of needle puncture and catheter insertion. Therefore, ultrasound can increase the chances of success during the procedure and reduces the complication rates [7,18]. Moreover, fluoroscopy can be used to control any guidewire kinking or vessel perforation during the insertion of a dialysis catheter, thus preventing many, if not all, complications to a definite degree [7]. Although a venogram is an invasive procedure, performing it in a suspected patient with complications usually enables instant imaging of the venous injury site. Post-procedural imaging (CXR, CT scan, or ultrasonogram) is recommended for the detection of such complications [2,13], especially when the patient complains of retrosternal chest pain and respiratory distress during catheter placement or aspiration of blood [8]. Injuries sometimes may lead to cardiac tamponade and median sternotomy alone is adequate to expose an innominate-caval confluence. However, for adequate exposure of the subclavian-internal jugular junction, the incision may be extended into the neck along with resection of the clavicle. Clamps, ligations, or rarely, a cardiopulmonary bypass is necessary to achieve control of bleeding. Mediastinal hematoma, if otherwise not symptomatic, can be conservatively managed [4,14].

Common popular treatment options are surgical repair by VATS [5-7] and endovascular repair, embolization, balloon tamponade, etc. [2,8,9,13]. In certain cases like ours, emergency thoracotomy or median sternotomy may be needed to remove blood clots, manage hematoma, and repair vascular tears [13].

## Conclusions

An inadvertent brachiocephalic vein injury due to the introduction of a large caliber dilator of the dialysis catheter can end with serious vascular injuries. We recommend the use of real-time fluoroscopy and/or ultrasonography during tunneled dialysis catheterization to prevent such complications, though image guidance cannot replace the meticulousness of the performer. A safer dilator design and sound insertion method have been suggested for the prevention of such complications, and a timely open surgical procedure can save patients' lives in most cases.
